# Heart failure patients’ experiences with continuity of care and its relation to medication adherence: a cross-sectional study

**DOI:** 10.1186/1471-2296-13-86

**Published:** 2012-08-20

**Authors:** Annemarie A Uijen, Marije Bosch, Wil JHM van den Bosch, Hans Bor, Michel Wensing, Henk J Schers

**Affiliations:** 1 Radboud University Nijmegen Medical Centre, Department of Primary and Community care 117, PO Box 9101, 6500, HB, Nijmegen, The Netherlands; 2 Radboud University Nijmegen Medical Centre, IQ Healthcare, PO Box 9101, 6500, HB, Nijmegen, The Netherlands

**Keywords:** Continuity of care, Personal continuity, Team continuity, Cross-boundary continuity, Heart failure, Medication adherence

## Abstract

**Background:**

A growing number of health care providers are nowadays involved in heart failure care. This could lead to discontinuity and fragmentation of care, thus reducing trust and hence poorer medication adherence. This study aims to explore heart failure patients’ experiences with continuity of care, and its relation to medication adherence.

**Methods:**

We collected data from 327 primary care patients with chronic heart failure. Experienced continuity of care was measured using a patient questionnaire and by reviewing patients’ medical records. Continuity of care was defined as a multidimensional concept including personal continuity (seeing the same doctor every time), team continuity (collaboration between care providers in general practice) and cross-boundary continuity (collaboration between general practice and hospital). Medication adherence was measured using a validated patient questionnaire. The relation between continuity of care and medication adherence was analysed by using chi-square tests.

**Results:**

In total, 53% of patients stated not seeing any care provider in general practice in the last year concerning their heart failure. Of the patients who did contact a care provider in general practice, 46% contacted two or more care providers. Respectively 38% and 51% of patients experienced the highest levels of team and cross-boundary continuity. In total, 14% experienced low levels of team continuity and 11% experienced low levels of cross-boundary continuity. Higher scores on personal continuity were significantly related to better medication adherence (p < 0.01). No clear relation was found between team- or cross-boundary continuity and medication adherence.

**Conclusions:**

A small majority of patients that contacted a care provider in general practice for their heart failure, contacted only one care provider. Most heart failure patients experienced high levels of collaboration between care providers in general practice and between GP and cardiologist. However, in a considerable number of patients, continuity of care could still be improved. Efforts to improve personal continuity may lead to better medication adherence.

## Background

Heart failure is a chronic disease with a high prevalence, reaching 1-2% in western countries [1]. Its burden is expected to rise in the coming decades due to an ageing population and longer survival from cardiovascular disease [1,2]. Heart failure is associated with major morbidity and high health care costs, due to high hospital admission rates [3].

Heart failure patients, among other patients with a chronic disease, are known to value continuity of care, in particular seeing the same doctor (personal continuity) [4]. Having a personal care provider is related to more confidence in the care provider [5,6], more patient satisfaction [6,7], increased feelings of being helped forward [6] and higher quality of patient’s life [8,9].

However, for patients with a chronic disease, recent developments in care can result in lack of continuity. An increasing number of care providers is nowadays involved in their care. Many patients contact several general practitioners (GPs) in one practice and probably also contact several specialists in one department. In addition, nurse physicians and practice nurses are often involved in the care for chronically ill patients. This could lead to discontinuity and fragmentation of care, which in turn may reduce trust and result in poorer medication adherence [10,11]. Communication and cooperation between all providers involved becomes increasingly important to guarantee continuity [12-15].

Patients experiences with personal continuity are well researched. However, little is known about patients experiences with communication and cooperation between care providers in general practice (team continuity) and between general practice and specialist care (cross-boundary continuity).

The aim of this article is therefore to analyse the degree to which heart failure patients contact their usual care provider (personal continuity) and the degree of communication and cooperation between several care providers involved in the care for these heart failure patients (team and cross-boundary continuity). We will also analyse the relation between continuity of care and patients’ medication adherence, for which the evidence is still inconclusive [10,11].

## Methods

### Participants

In the Netherlands, every patient is enlisted with a GP who functions as a gatekeeper for specialist care. Most heart failure patients in the Netherlands obtain the medical care for their heart failure by their GP on their own initiative. Nurse practitioners have more and more taken over the monitoring of blood pressure and other risk factors.

In the period 2005–2006, we invited 415 general practices in 15 different hospital regions to participate in this study of which 72 GPs in 42 practices agreed to participate. These practices are representative for Dutch practices regarding urbanization rate and type of practice. All patients with a diagnosis of chronic heart failure, according to their GP, were eligible to be included. We excluded patients with a terminal disease, a mental impairment, Dutch language problems or when the GP decided that patients should not be included in the study for other reasons. The study is powered on the primary study outcome ‘health related quality of life’. This article focuses on continuity of care and medication adherence as outcome measures, for which we performed no power calculations. Ethical permission for the study was obtained from the ethics committee Arnhem-Nijmegen. More detailed information regarding this study has been previously published [16].

### Measurements

#### Continuity of care

We measured the experienced continuity of care using a previously developed questionnaire (see Table 1 for specific items). This questionnaire is based on 30 patient interviews that were conducted as part of a study on continuity of care and consists of 11 items [17]. The questionnaire was tested among six GPs/senior researchers and eight patients (content validity) to make sure no significant items were missing and all items were understood correctly. After the test phase, minor changes to the questionnaire were made. 

**Table 1:** Results of items measuring continuity of care

***Personal continuity in general practice***	***0***	***1***	***2***	***3***	***4 or more***
1. How many different care providers have you seen in general	161 (53.1%)	76 (25.1%)	42 (13.9%)	19 (6.3%)	5 (1.7%)
practice in the last year for your heart disease? (n = 303)					
2. Total number of contacts with general practice in last year	10 (4.1%)	9 (3.7%)	14 (5.7%)	16 (6.5%)	196 (80.0%)
(according to medical record) (n = 245)					
***Team continuity in general practice***	*Never*	*Seldom*	*Sometimes*	*Often*	*Always*
1. The treatment of my heart disease in general practice	25 (10.0%)	9 (3.6%)	19 (7.6%)	43 (17.3%)	153 (61.4%)
goes smoothly (n = 249)					
2. The care of the different care providers in general practice	21 (8.2%)	11 (4.3%)	18 (7.1%)	63 (24.7%)	142 (55.7%)
for my heart disease is connected (n = 255)					
3. The care providers in general practice often give me	129 (49.8%)	79 (30.5%)	29 (11.2%)	8 (3.1%)	14 (5.4%)
contradictory advice about my heart disease (n = 259)					
4. The care providers in general practice involved in the care	21 (8.2%)	7 (2.7%)	24 (9.3%)	64 (24.9%)	141 (54.9%)
for my heart disease communicate well with each other (n = 257)					
5. The care providers in general practice involved in the care	12 (4.7%)	11 (4.3%)	31 (12.2%)	54 (21.2%)	147 (57.6%)
for my heart disease have knowledge of my medical record (n = 255)					
6. The care providers in general practice involved in the care	20 (7.9%)	12 (4.7%)	27 (10.7%)	50 (19.8%)	144 (56.9%)
for my heart disease have knowledge of previous visits (n = 253)					
***Cross-boundary continuity***	*Never*	*Seldom*	*Sometimes*	*Often*	*Always*
1. The care of the GP and the cardiologist is connected (n = 191)	9 (4.7%)	12 (6.3%)	11 (5.8%)	38 (19.9%)	121 (63.4%)
2. The GP and the cardiologist often give me contradictory	110 (56.4%)	48 (24.6%)	22 (11.3%)	9 (4.6%)	6 (3.1%)
advice about my heart disease (n = 195)					
3. The GP and the cardiologist transfer information about	9 (4.6%)	11 (5.6%)	14 (7.2%)	37 (19.0%)	124 (63.6%)
my heart disease well between each other (n = 195)					
4. The GP and the cardiologist communicate well (n = 190)	12 (6.3%)	12 (6.3%)	17 (8.9%)	40 (21.1%)	109 (57.4%)

GP: General practitioner; Items are measured using a patient questionnaire unless otherwise indicated.

Three dimensions of continuity can be identified in the questionnaire, corresponding to the literature [12,15,18]:

1. Personal continuity (1 item): the number of care providers (GPs and/or nurses) that patients saw in general practice for their heart disease in the last year.

To further analyse this dimension of continuity of care, we also reviewed the patients’ medical records for the total number of contacts with the general practice in the last year. Due to limited resources, the collection of medical record data was limited to a random sample of maximum 15 patients per practice.

2. Team continuity in general practice (6 items): the extent to which care providers (GPs and/or nurses) in general practice have knowledge of the patient and communicate and cooperate with each other.

3. Cross-boundary continuity (4 items): the extent to which GP and cardiologist communicate and cooperate with each other.

For the domains 2 and 3, responses were recorded on a five-point scale (1 = never, 5 = always). We tested the questionnaire in a sample of Dutch patients with COPD, heart failure or a mental illness and subsequently performed principal factor analysis on the 10 items of domain 2 and 3, which confirmed the two presumed factors (construct validity). The eigenvalues of both factors were 8.064 (team continuity) and 1.135 (cross-boundary continuity). The two factors together explained a total variance of 83.6% (respectively 42.7% and 40.9%).

#### Medication adherence

We measured patients’ medication adherence by using the validated measure of Morisky et al. [19]. This scale measures self-reported intentional (two items) and unintentional (two items) non-adherence to medicines. The items can be answered on a 5-point Likert scale ranging from 1 (no adherence) to 5 (full adherence). Medication adherence was measured as the sum score of the four questions, varying from 4 to a maximum of 20.

Both (anonymous) questionnaires were sent simultaneously by mail. In case of non-response, a reminder was sent after three to four weeks.

### Analysis

First, we explored the results on the items measuring personal, team and cross-boundary continuity. We excluded cases in which half or more of the questions of a specific factor were missing, i.e. 3 or more questions on team continuity or 2 or more questions on cross-boundary continuity. All remaining missing values were imputed by patient’s mean of the non-missing items on the specific factor. The answers on the negatively keyed questions were then recoded.

The total score of team continuity ranged from 6 to a maximum of 30 and the total score of cross-boundary continuity ranged from 4 to a maximum of 20. Due to the wide range in total scores, we sub-classified the total score of team and cross-boundary continuity into 5 subcategories. These categories consisted of the maximum score and subsequently 4 subcategories with an equal range. For team continuity: (1) 30 (max), (2) 24–29, (3) 18–23, (4) 12–17 and (5) 6–11. For cross-boundary continuity: (1) 20 (max), (2) 16–19, (3) 12–15, (4) 8–11 and (5) 4–7.

We sub-classified the total score of team and cross-boundary continuity into 5 subcategories: the maximum score and subsequently 4 equal subcategories.

All analyses were performed using SPSS 16. We analysed the relation between continuity of care and patients’ medication adherence by using chi-square tests. A p-value <0.05 was considered statistically significant.

Analyses were performed both including and excluding the patients that did not have contact with any care provider in general practice in the last year for their heart failure. As the results in both analyses did not differ, we present the analyses including these patients in this article.

## Results

We sent the questionnaire measuring continuity of care to 461 patients. In total, 370 patients (80%) responded. We excluded 43 patients due to missing values (3 or more questions on team continuity or 2 or more questions on cross-boundary continuity). Consequently, we analysed data from 327 patients. Most patients were Dutch (93%), above 70 years of age (73%) and could be classified as NYHA class I (49%). Patients’ sex was equally distributed (Table 2).

**Table 2:** Characteristics of the study population (n = 327)

**Age, mean (SD)**	**74.9 (10.1)**
<65	54 (16.5%)
65-70	33 (10.1%)
71-75	59 (18.0%)
76-80	78 (23.9%)
81-85	62 (19.0%)
>85	41 (12.5%)
**Sex**	
Male	164 (50.2%)
Female	163 (49.8%)
**Nationality**	
Dutch	305 (93.3%)
Other	12 (3.7%)
Missing	10 (3.1%)
**NYHA class**	
Class I	159 (48.6%)
Class II	72 (22.0%)
Class III	87 (26.6%)
Class IV	7 (2.1%)
Missing	2 (0.6%)

SD: standard deviation, NYHA: New York Heart Association.

### Levels of experienced continuity

Table 1 shows the results of the items measuring continuity of care. A total of 53% of patients stated not seeing any care provider (GP or nurse) in general practice in the last year for their heart failure. In total, 54% of these patients that did not contact their general practice, did contact a cardiologist in the last year. Based on the medical record review, we found that only 10 patients (4.1%) did not have any contact with a care provider in general practice at all in the last year.

In total, 25% saw one care provider in general practice for their heart failure, 14% saw two care providers and 8% of patients saw 3 or more care providers in general practice.

The questions concerning team continuity and cross-boundary continuity mostly scored positive: each item scored maximum by at least half of the patients.

Table 3 shows the total score of team and cross-boundary continuity. Almost 38% of patients experienced maximum team continuity (score 30), while 51% of patients experienced maximum cross-boundary continuity (score 20). Respectively 14% and 11% of patients experienced very low levels of team and cross-boundary continuity (total score less than 18 or 12, respectively).

**Table 3:** Total score of team and cross-boundary continuity

***Team continuity in general practice (n = 261)***	
30	99 (37.9%)
24-29	94 (36.0%)
18-23	32 (12.3%)
12-17	18 (6.9%)
6-11	18 (6.9%)
***Cross-boundary continuity (n = 195)***	
20	99 (50.8%)
16-19	50 (25.6%)
12-15	24 (12.3%)
8-11	12 (6.2%)
4-7	10 (5.1%)

The score of team continuity varied between 6 (minimum) and 30 (maximum).

The score of cross-boundary continuity varied between 4 (minimum) and 20 (maximum).

### Continuity and medication adherence

In total, 74% of patients were fully adherent (score 20), 15% scored 19 points on the medication adherence measure, 7% scored 18 points and 5% scored 17 points or less.

Table 4 shows the relation between experienced continuity of care and patients’ medication adherence. Patients who saw three or more care providers in general practice were less likely to be fully adherent than patients who saw less care providers (p < 0.01). We found a non-linear relation between experienced team continuity and medication adherence: both high and low levels of team continuity were associated with maximum medication adherence, while the mid-levels of team continuity were associated with the lowest medication adherence (p = 0.04). No relation was found between cross-boundary continuity and medication adherence.

**Table 4:** Relation between continuity of care and medication adherence

	**Medication adherence**			
	**17 or less**	**18**	**19**	**20 (maximum)**
***Personal continuity in general practice (p < 0.01)***				
0 care providers	4 (2.6%)	5 (3.2%)	28 (17.9%)	119 (76.3%)
1 care provider	9 (12.0%)	6 (8.0%)	5 (6.7%)	55 (73.3%)
2 care providers	1 (2.4%)	5 (12.2%)	4 (9.8%)	31 (75.6%)
3 or more care providers	0 (0.0%)	4 (16.7%)	4 (16.7%)	16 (66.7%)
***Team continuity in general practice (p = 0.04)***				
30	3 (3.0%)	4 (4.0%)	11 (11.1%)	81 (81.8%)
24-29	5 (5.3%)	8 (8.5%)	15 (16.0%)	66 (70.2%)
18-23	3 (9.7%)	6 (19.4%)	6 (19.4%)	16 (51.6%)
12-17	3 (17.6%)	0 (0.0%)	1 (5.9%)	13 (76.5%)
6-11	0 (0.0%)	2 (11.8%)	3 (17.6%)	12 (70.6%)
***Cross-boundary continuity (p = 0.19)***				
20	2 (2.1%)	3 (3.1%)	14 (14.6%)	77 (80.2%)
16-19	3 (6.1%)	4 (8.2%)	10 (20.4%)	32 (65.3%)
12-15	1 (4.3%)	3 (13.0%)	2 (8.7%)	17 (73.9%)
8-11	0 (0.0%)	3 (25.0%)	1 (8.3%)	8 (66.7%)
4-7	1 (11.1%)	0 (0.0%)	1 (11.1%)	7 (77.8%)

Medication adherence varied between 4 (minimum) and 20 (maximum). Because of the small number of patients scoring 17 or less, we grouped these patients together.

## Discussion

Based on a self-report, we found that more than half of the patients did not contact any care provider in general practice for their heart failure in the last year, though half of them did have contact with a cardiologist. About half of the patients who did contact general practice, had contact with two or more care providers. Most patients experienced acceptable levels of communication and cooperation between care providers in general practice and between GP and cardiologist, while 10-15% experienced very low levels.

Medication adherence was significantly less in patients who saw three or more care providers. Patients who did not contact any care provider at all in the last year had high levels of medication adherence. Team continuity was related to medication adherence, but in a non-linear way. No relation was found between cross-boundary continuity and medication adherence (p = 0.19).

### Implications for practice and research

We found that better personal continuity is also related to better medication adherence. Better medication adherence may lead to lower hospitalization rates, lower morbidity and mortality and lower health care costs [20-23]. Most Dutch heart failure patients experience high levels of personal, team and cross-boundary continuity of care. However, in a considerable amount of patients, personal continuity can be improved in order to achieve more confidence in the care provider, more patient satisfaction and higher quality of patient’s life.

A possible explanation for the relation between personal continuity and medication adherence could be that patients have less trust in care providers when contacting more care providers [24]. Nowadays, an increasing number of care providers work part-time, thus making it more likely for a patient to have contact with more than one care provider. Nevertheless, we think personal continuity can still be improved as most contacts are non-urgent, making it possible to make an appointment with their own care provider.

When more care providers are involved in the care of a patient, we found that team and cross-boundary continuity can be improved in at least 10-15% of patients. Team and cross-boundary continuity can for example be improved by better communication between care providers (e.g. better registration in the electronic medical record and faster communication by mail between general practitioner and specialist).

The importance of the non-linear relationship between team continuity and medication adherence is unknown. It should be interpreted with caution. More research is needed before firm conclusions can be drawn about this relationship.

### Comparison with previous studies

Previous studies found comparable levels of experienced continuity of care [6,25-27].

We found only two studies investigating the relation between personal continuity and medication adherence. One of them found a positive relation between seeing the care provider who prescribed the medication and medication adherence [10], while the other study found no relation between the proportion of consultations with the usual physician and medication adherence [11]. We found no studies investigating the relation between team or cross-boundary continuity and medication adherence.

### Limitations

One of the limitations of this study is that 53% of patients answered that they did not see any care provider in general practice in the last year for their heart failure. However, most of these patients did fill in the other continuity questions, which can make the reliability of the answers doubtful. We made the assumption that most of the patients stating not have seen any care provider in general practice in the last year for their heart failure, saw their GP for other disorders in that year during which the GP also monitored their heart failure (e.g. blood pressure). This assumption is strengthened by the fact that, based on the medical record search, only 10 patients (4.1%) did not have any contact at all with a care provider in general practice in the last year. The results of the analyses of the data including and excluding these patients did not differ.

Another limitation is the cross-sectional design of this study. As a consequence, we can only hypothesize about the causality of the relation between continuity and medication adherence and our results should be interpreted with caution.

A last limitation is that the study is powered on the outcome measure ‘health related quality of life’. Possibly, too few patients were included to find a relation between team- or cross-boundary continuity and medication adherence.

## Conclusions

A small majority of patients that contacted a care provider in general practice for their heart failure, contacted only one care provider (personal continuity). Most heart failure patients experienced high levels of collaboration between care providers in general practice (team continuity) and between GP and cardiologist (cross-boundary continuity). However, in a considerable amount of patients continuity can still be improved. Efforts to improve personal continuity may lead to better medication adherence.

## Competing interests

The author declares that they have no competing interests.

## Authors’ contributions

AU participated in data collection, analysis and interpretation of the data and drafted the manuscript, MB participated in data collection and the design of the study, WvdB participated in analysis and interpretation of the data, HB participated in the design of the study and performed the statistical analysis, MW participated in data collection and the design of the study, HS participated in the design of the study, analysis and interpretation of the data and helped to draft the manuscript. All authors read and approved the final manuscript.

## Author’s information

AU, WvdB and HS are general practitioners, HB is a statistician, AU is also a PhD graduate. MB, MW and HS have a PhD and WvdB is professor of general practice.

## Pre-publication history

The pre-publication history for this paper can be accessed here:

http://www.biomedcentral.com/1471-2296/13/86/prepub
